# EXPLORING THE MODERATING ROLE OF COGNITIVE FUNCTION IN SCREEN TIME AND STRESS

**DOI:** 10.1093/geroni/igae098.4356

**Published:** 2024-12-31

**Authors:** Nelson Roque, Robert McGill

**Affiliations:** Pennsylvania State University, University Park, Pennsylvania, United States; Pennsylvania State University, University Park, Pennsylvania, United States

## Abstract

Understanding the interplay between brain health and digital media use is crucial in our current technological era. Prior research has shown that stress influences screen time usage and that screen time can impact cognitive function and brain structure (Neophytou, E., Manwell, L.A., & Eikelboom, R., 2021). This online survey study, conducted on Prolific, examines the relationship between screen time and stress, as moderated by momentary cognitive functioning, in a sample of 319 participants (mean age = 40.5 years, age range 19-77, 61% female). Momentary cognitive processing speed was assessed using a web-based cognitive assessment developed by M2C2 (NIA #U2CAG060408), stress via the Perceived Stress Scale, and screen time through self-reported data (yesterday’s screen time reported on cell phone). The bootstrapped SEM moderation analysis found that stress significantly increases screen time (stress_effect = 0.169, p = 0.004, 95% CI [0.051, 0.287]). The interaction effect (interaction_effect = 0.153, p = 0.011, 95% CI [0.031, 0.278]) indicates that faster processing speed (better cognition) amplifies the impact of stress on screen time. For example, in moments when one has faster processing speed, stress may lead to increased screen use as a way to cope with heightened awareness of stressors. Cognition alone does not significantly affect screen time (cognition_effect = 0.022, p = 0.740, 95% CI [-0.102, 0.162]). These findings underscore the need to consider cognitive dynamics when studying the relationship between digital media use and well-being. Future research should employ real-time data collection to explore how cognitive changes (across domains), affect screen time behaviors.

